# Most Americans support minimizing administrative burdens for Medicaid recipients as the public health emergency ends

**DOI:** 10.1093/haschl/qxad001

**Published:** 2023-06-20

**Authors:** Simon F Haeder, Donald P Moynihan

**Affiliations:** Department of Health Policy & Management, Texas A&M University, 1266 TAMU, 212 Adriance Lab Road, College Station, TX 77843, United States; McCourt School of Public Policy, Georgetown University, 37th Street NW O Street NW, Old North #100, Washington, DC 20057, United States

**Keywords:** Medicaid, public health emergency, COVID-19, access to care

## Abstract

During the coronavirus disease 2019 (COVID-19) public health emergency (PHE), states were barred from disenrolling anyone from Medicaid unless the beneficiary asked to be disenrolled, moved out of state, or died. Coverage increased, but as the PHE ends an estimated 7 million eligible Americans are expected to lose insurance due to difficulty navigating the renewal process. The end of the PHE therefore offers state policymakers a chance to reassess the value of such administrative burdens as a variety of policy tools are available to mitigate these losses. We inform this discussion via a national survey that captures public preferences around administrative burdens in public health insurance. We find strong public support for burden-reduction techniques that minimize coverage losses such as using administrative data to shift burdens onto the state and better outreach and communication, with an average of 74% of respondents supporting each policy tool. This support holds across the ideological spectrum and demographic groups, but it is stronger among liberals than conservatives, for those with more direct experience of burdens, those who struggle with such burdens, and for those with lower racial prejudice.

## Introduction

Administrative rules and requirements are a basic component of a modern welfare state around the world,^1^ justified as ensuring standardized treatment and minimizing fraud by limiting access to those eligible.^2^ However, at times, such requirements may evolve into an unreasonable burden that prevent eligible individuals receiving benefits that they are legally entitled to.^3-7^ As a result, key public services may not reach the target population, becoming less effective while imposing substantial costs on the individuals affected. While complaints about administrative frustrations may be common, we know little of how the public thinks about efforts to reduce burden.

Administrative requirements and their policy implications have gained significant attention over the last several years due to the coronavirus disease 2019 (COVID-19) pandemic, which posed significant health and policy challenges for governments worldwide. In the United States, as elsewhere, the pandemic generated extraordinary policy experimentation, causing citizens and policymakers alike to reexamine some core assumptions about not just the type of policies that are needed but how they are delivered. Unsurprisingly, much innovation occurred in the domain of health policy.^8,9^

Two of the primary vehicles for change were the Families First Coronavirus Response Act (FFCRA)^10^ as well as repeated declarations of public health emergency (PHE) by both the Trump and Biden Administrations.^11^ The PHE allows the federal government to take certain discretionary policy actions that would otherwise not be allowed under federal law.^12^ In response to the substantial needs that Americans incurred as a result of the pandemic, federal and state governments sought to reduce the burdens associated with health insurance enrollment and maintenance. A particular focus has been on the Medicaid program, the US program for low-income individuals and families as well as children, pregnant women, and eligible aged, blind, or disabled people whose income is insufficient to meet the cost of necessary medical services.^13^ For example, as a condition of increased COVID-19 federal matching funds for Medicaid, states were barred from reducing eligibility, making enrollment processes more difficult, or involuntarily disenrolling anyone for the duration of the PHE.^14,15^ As a result, public health insurance climbed to record levels, with a 30% increase in Medicaid coverage alone.^11^

As the PHE expires, state policymakers are poised to redeploy a series of administrative burdens by asking people to navigate complex bureaucracy, fill out confusing paperwork, track down documentation, or spend more resources to travel to appointments or paying for help filling out forms.^7^

The end of the PHE has two potential effects. First, many eligible individuals will lose public health insurance they are entitled to, as overwhelmed states and the public come to terms with unfamiliar processes and workloads.^16^ More than 7 million Americans are predicted to lose insurance coverage due to the difficulty navigating the renewal process and other administrative issues despite being eligible for public coverage either in the Medicaid program itself or the Affordable Care Act (ACA) marketplaces.^17^

The second potential effect of the end of the PHE will be to recalibrate access to public health insurance programs. Will states revert to the old administrative processes they had in place prior to the PHE and reimpose burdens on potential beneficiaries? Such processes led to a decline in insurance coverage for children in the years immediately before the pandemic.^18^ Or will states adopt evidence-based tools^5^ that make access easier, by shifting burdens away from citizens and improving outreach?

At this point, it is unclear which of these two potential effects will prevail. On the one hand, states have been required to plan for the unwinding, but were allowed significant discretion, especially in the long run. In late 2022, Congress passed an omnibus bill that would allow states to begin processing Medicaid redeterminations by April 1st, phasing out the increased matching rate until the end of 2023.^11^ States have been encouraged by the federal government to use techniques to reduce burdens, by, for example, using administrative data to automatically re-enroll people who are clearly eligible (“ex parte processes”).^19^ On the other hand, much remains to be done. For example, the Kaiser Family Foundation reported that only eleven states were completing 50% or more of renewals using ex parte processes.^14,15^ To support states in these efforts, the new legislation maintains incentives in place to encourage states to limit burdens, ensuring they have up-to-date contact information, engage in active outreach before disenrolling anyone, and not make eligibility or processes more restrictive. Crucially, a recent study indicates that almost two in three adults with family Medicaid enrollment were unaware of the resumption of Medicaid redeterminations.^20^

As states choose how they manage the unwinding of the PHE, it is important to better understand how Americans think about these policy actions. While there is much evidence about public support for health policies and eligibility, we know little about public attitudes about administrative barriers that are consequential to access. Does the public want a return to the status quo, or do they support more active efforts to shift burdens away from citizens? To address this question, and to understand whether raising the salience of certain aspects of the PHE affects American public opinion, we fielded a large (*N* = 4157) preregistered national survey in late 2022 and early 2023. The survey queried respondents about nine specific policies intended to reduce burdens in the wake of the PHE that also included a survey experiment highlighting various detrimental effects of the unwinding.

## Administrative burdens and health

Administrative burdens are the frictions that people encounter in the implementation of public services, which can include learning about such administrative processes, completing forms and documentation requirements, and the associated psychological costs.^3^ Such frictions can play a significant role in limiting access to public services.^21,22^ Burdens can harm health in multiple ways, most obviously by restricting access to the benefits of health-protective programs, such as health insurance or safety net supports,^23,24^ or health services.^25^

One particular claim of administrative burden theory is that as burdens are costly to overcome—requiring not just time but also financial resources, cognitive skills, or administrative literacy—they represent a bigger barrier for those with fewer resources and thus disproportionately hurt the disadvantaged.^26^ As a result, burdens can exacerbate underlying sources of inequality.^27^ For example, burdens that saw lower enrollment for children in public insurance had disproportionate effects on families where parents lacked a college degree or held Hispanic or immigrant status.^9^ Burdens have also long been associated with reduced enrollment in programs like Medicaid,^28,29^ and the Children’s Health Insurance Program (CHIP).^30^ They can also explain highly divergent take-up rates of benefits that exacerbate societal inequities.^31-33^

Importantly, such administrative burdens can be opaque because they are complex, dull, and difficult to understand both in terms of their processes and outcomes. This allows them to serve as a form of “policymaking by other means”: policymakers sometimes allow such burdens to exist because of a lack of understanding of their impact, or even actively use them to limit access in ways that are less visible and politically costly than changes to program eligibility.^3^ Changes that reduce eligibility generally require legislation, drawing public attention, if not opposition, and with little doubt about their effects. By contrast, the public is less likely to be attentive to technical details during implementation, such as longer forms, mail-based renewal processes, or requirements for documentation, which nonetheless can carry with them substantial consequences.

Lack of systematic analytical attention to burdens in policy analysis, media coverage, or political debate helps to explain why such hassles can be both widely acknowledged—most of us have a story of dealing with frustrating government processes—but nonetheless remain unaddressed. Without such attention, it is easier for policymakers to assume that the public or users accept or even condone existing processes as well as ensuing outcomes. Dysfunctional governmental administrative processes can thus remain in place, limiting the effectiveness of policies and, importantly, deny benefits to individuals who are technically eligible for them.

This may be starting to change. The topic of administrative burden has become one of growing interdisciplinary interest, especially in the context of safety net programs.^34^ Media attention to the “time tax” that burdens create has grown.^35^ In addition, the Biden Administration has issued multiple executive orders that direct attention to the topic, identifying them as a source of inequality that federal agencies should better oversee.^36,37^ Governmentwide guidance, such as updated guidance of the Paperwork Reduction Act,^38^ provides explicit direction to agencies to actively seek out opportunities to identify and reduce burdens.

While developing a stock of knowledge about the existence of burdens, their effects, and alternative approaches represents one part of the policymaker toolbox, the deployment of such knowledge still depends upon public and policymaker views about burdens, the topic of our analysis. Of course, certain burdens are inevitable, while others serve “essential goals such as preventing fraud, reducing agency error rates, and rationing scarce products or services.”^2^ Yet, as the existing literature indicates, a substantial part of existing burdens fails to increase program integrity and merely impedes access to services.

## Study data and methods

### Study sample

In order to explore public opinion on how states may approach the unwinding of the PHE, we programed an original, preregistered survey in Qualtrics and fielded it via Lucid among a national sample of US adults (*N* = 4157) between December 21, 2022, and January 4, 2023. Lucid provides a national sample that closely approximates representativeness on key demographics like race, age, sex, income, and census region, and has been deemed an appropriate tool for survey research.^39^ To further improve representativeness, we weighted responses on gender, race, income, and education based on the US Census Current Population Survey to obtain population average treatment effects (see supplementary exhibits A1 & A2 in Appendix). A total of 7805 respondents initiated the survey. Of these, 7360 (94%) consented to take the survey after the survey introduction. The survey also included two standard attention checks to ensure data quality. Ultimately, 4157 respondents completed the survey (56%). The experiment received approval from the Institutional Review Boards at the appropriate institutions.

### Survey instrument

Before querying respondents about their opinion on various policies that reduce administrative burdens as related to unwinding of the PHE, we also included a survey experiment that raised the salience of various detrimental effects of this unwinding for respondents. Specifically, in addition to a control group with no treatment, we primed respondents for one of the following effects (For details on treatment, please see supplementary exhibits A4 & A5 in Appendix):

- overall coverage losses;
- disproportionate coverage losses for racial and ethnic minorities;
- coverage losses exacerbating racial inequities and systemic racism;
- disproportionate coverage losses for those with the lowest income;
- broader community effects of coverage losses.

After the treatments, we queried respondents about nine evidence-based policies to reduce administrative burdens, and thus coverage losses, as the United States transitions out of the PHE. (For details on the questions, please see supplementary exhibit A3 in Appendix). We organized these administrative actions that are related to burdens into two distinct categories. The first set of policies *shifts burdens to the state* and thus away from (potential) beneficiaries.^40^ Such actions seek to minimize compliance costs imposed on the public by tasking the state with taking a more proactive approach. These include tools like (1) automatic (ex parte) renewals, (2) using prefilled forms, (3) ensuring the most recent contact information from other programs (such as the Supplemental Nutrition Assistance Program [SNAP]) or sources (the US Post Office), (4) improving account transfer processes with ACA marketplaces, and (5) ensuring enough administrative capacity to perform such tasks. Such actions imply the active use of administrative data rather than compelling users to provide such data. The second general strategy is improved *outreach and communication*. This involves (6) resorting to nontraditional sources of communications like text messaging, (7) sending materials in plain language to potential enrollees, (8) increasing outreach and enrollment efforts, and (9) providing clear information about termination and enrollment procedures and how to get help if needed. Such actions generally reduce learning costs for beneficiaries. In all cases, respondents were asked whether states should adopt the burden-reduction policy or whether to return to the pre-pandemic status quo. We note that all these policy choices are focused on either reducing workload for potential beneficiaries or lowering information costs. None of the options are particularly likely to increase benefit fraud or misconduct.

This setup allows us to test whether Americans are generally supportive of reductions in administrative burdens as well as the effects of respondents’ ideology, racial prejudice, and experience with administrative burdens in their own lives. Moreover, we are able to test whether emphasizing the effects of failure to mitigate coverage losses exerts an effect on respondents’ perceptions of administrative burdens. The results offer an unprecedented look at public opinion as it relates to how states restructure access to health insurance in the aftermath of the pandemic, especially on the question of whether the public, and which part of the public, and under what conditions, prefers burdens versus tools to simplify access.

### Hypotheses

Much of the academic literature has focused on the incidence of administrative burdens as well as their potentially harmful effects on (potential) beneficiaries. At the same time, knowledge about the general public's tolerance of burdens for programs like Medicaid is limited.^41^ However, while Americans are generally concerned about the potential for fraud in public-assistance programs, all policies presented to respondents are rather unlikely to increase fraud, as described in further detail elsewhere here. Moreover, we particularly expect that the dramatic impact of the pandemic on Americans’ lives further increased respondents’ tolerance for administrative easings (We also note that we presented the questions to respondents as asking them whether they would like to continue to the post-COVID 19 policy or revert back to the status quo ex ante. See supplementary exhibit A3 in Appendix for details). We hypothesize the following:

#### Hypothesis 1: Respondents will favor policies to reduce administrative burdens rather than return to pre-PHE policies.

Prior research also suggests that, in the domain of redistributive safety net policies, conservatives tend to be more supportive of burdens.^23^ Such ideological support for burdens reflects both higher opposition to such programs, plus related attitudes that also predict support for burdens, such as beliefs about the deservingness of clients, and concerns about fraud. We thus hypothesize the following:

#### Hypothesis 2: Liberals across all treatments will show higher support for easing administrative burdens than conservatives.

Beliefs about burdens reflect not just partisanship and ideology but also people's personal experiences with state interactions. For example, Danish policymakers who had relied on welfare benefits in the past were less supportive of burdens in welfare programs as legislators.^34^ Such direct experiences create policy feedback lessons, teaching clients about the relative difficulties that burdens create, and raising questions about their value. Americans who have relied on Medicaid and SNAP tend to be less supportive of burdens in such policies.^41^ We thus hypothesize the following:

#### Hypothesis 3: Those connected to the Medicaid program will be more supportive of easing administrative burdens.

People may also vary in terms of their administrative literacy,^42^ as reflected by their general ability to understand and manage administrative tasks. As people evaluate their administrative skills poorly, they are more likely to be wary of administrative burdens imposed upon themselves or others. We thus hypothesize the following:

#### Hypothesis 4. Those who struggle with completing administrative tasks will be more supportive of easing administrative burdens than those who do not.

Public opinion about public-assistance programs like Medicaid^43-46^ has long been found to be influenced by perceptions about race.^47-49^ The racialization of US health policy has only been further exacerbated in the wake of the election of President Obama^50^ as well as the implementation of the ACA.^51,52^ We thus hypothesize the following:

#### Hypothesis 5. Those with lower levels of prejudice towards minorities will be more supportive of easing administrative burdens than those with higher levels.

Lastly, behavioral economics has found strong evidence that individuals are often hesitant to surrender benefits once they have been achieved.^53,54^ We use experimental treatments to convey the negative effects of administrative burdens, using a variety of frames, including overall coverage losses, plus frames that emphasize race, class, and broader community effects. We thus hypothesize the following:

#### Hypothesis 6: Increasing the salience of administrative burdens by emphasizing losses will increase support for easing administrative burdens.

### Data analysis

We conducted two broad sets of analyses. First, we analyzed responses to the nine individual policies reducing burdens described above as the nation transitions out of the PHE. In order to facilitate presentation of the results, we also generated three scales. Our first scale combines all nine policies into an overall measure, while our second scale combines all policies focused on *shifting the burden to the state*; the third scale combines all policies focused on *outreach*.^42^ We generally present the findings from the analysis of these three scales below but note that results for the analysis of individual policies were essentially analogous throughout. Due to the survey design with one control group and five different treatments, we estimate a number of standard ordinary least squares (OLS) models to test our hypotheses described above. To assess the effect of ideology, we interacted indicator variables for each treatment with our three-category ideology variable (liberal, other, conservative). To assess whether a connection with the Medicaid program affects perceptions, we interacted an indicator variable that was coded 1 if the respondent had ever been on Medicaid and 0 otherwise. In order to test our third hypothesis whether those who struggle with completing administrative tasks will be more supportive of administrative easings, we split respondents into tertiles depending on the degree they struggle with the completion of administrative tasks and estimated models with indicators for each tertile. In order to determine the level of racial prejudice against minorities, we followed the standard practice to rely on three questions utilized in the American National Election Survey for Whites, Blacks, Hispanics, and Asians.^55^ We then created a scale that combined the three items (Standard tests for the scales indicating high reliability of the composite scores), split respondents into tertiles, and estimated models with indicators for each tertile. Last, to test whether increasing the salience of administrative burdens had an effect, we compared the results from each treatment with the control group. We utilized the results from the OLS estimates to estimate predictive means and compared differences using mlincom in Stata (StataCorp).^56^ Per our preregistration, we considered a *P* value lower or equal to .10 as statistically significant throughout our analyses.

## Study results

Given the opaque nature of administrative burdens, there is significant policy relevance in examining descriptive data in levels of support for burden-reduction efforts. To do so, we first present an overview of respondent preferences for each of the nine individual policies presented in the survey. In each case, subjects were asked to choose between a policy that would ease burden versus the pre-pandemic status quo. While burden-reduction efforts might seem more obviously attractive, given a well-established behavioral preference for status quo outcomes,^54^ it is not clear which outcome individuals would prefer.

**Figure 1.**
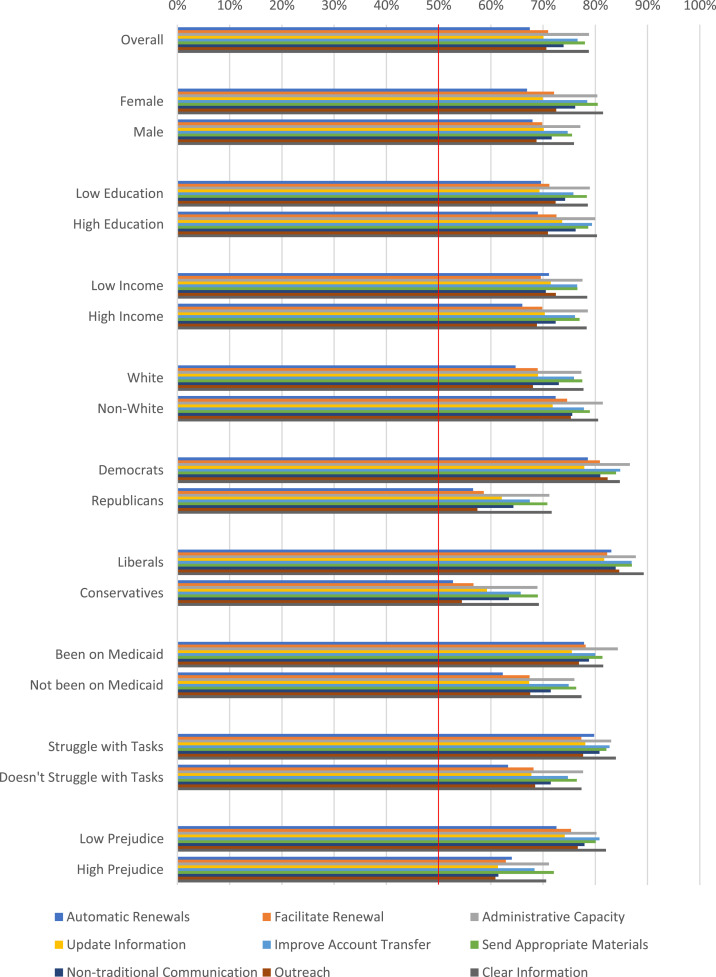
Proportion of respondents supportive of reducing administrative burdens.

We find broad support for reducing burdens across all groups and across all nine burden-reduction policies, with a mean level of support of 74% (Figure 1).^57^ Indeed, more than two-thirds of respondents supported the reduction of administrative burdens across the nine policies; for some policies, like improvements in account transfer to the ACA marketplaces as well as the provision of clear information about termination and enrollment procedures, support among respondents approached 80%. Support for all demographics presented in Figure 1 is larger than 0.500 (*P* < .05) with the exception of conservatives and automatic renewals (*P* < .16). Notably, respondents across a broad range of demographic subgroups based on education, race, income, ideology, partisanship, and previous experience with the Medicaid program show support in excess of 0.500 for every single policy we queried them about. These findings even hold for individuals with high levels of racial prejudice.

**Figure 2.**
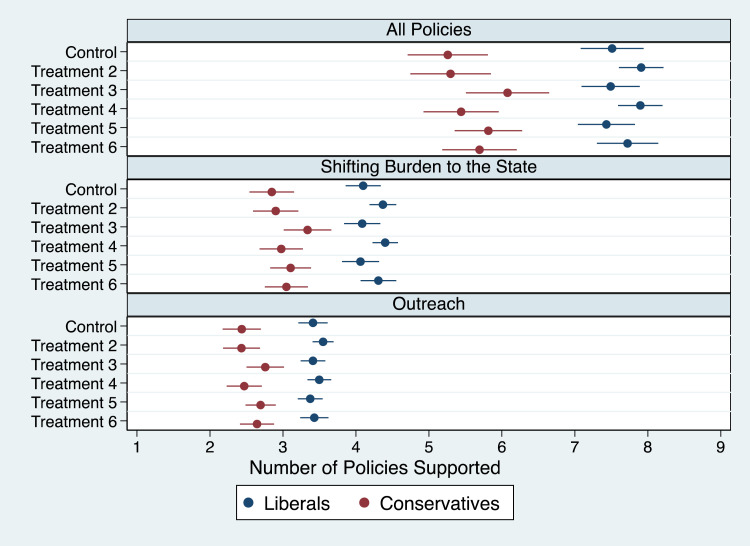
Mean number of policies supported by respondents, by partisanship.

We also found strong support for our second hypothesis (Figure 2) as liberals are consistently more supportive of administrative easings than conservatives across the three scales (*P* < .001). The findings are also substantively large, with liberals supporting between 7.4 and 7.9 policies (where nine is the maximum) and conservatives supporting between 5.3 and 6.1 policies. We note that these findings are also consistent across the scales focused on *shifting burdens to the state* and *outreach* as well as all nine individual policies and across all treatments (see supplementary exhibits A6 & A7 in Appendix).^58^

**Figure 3.**
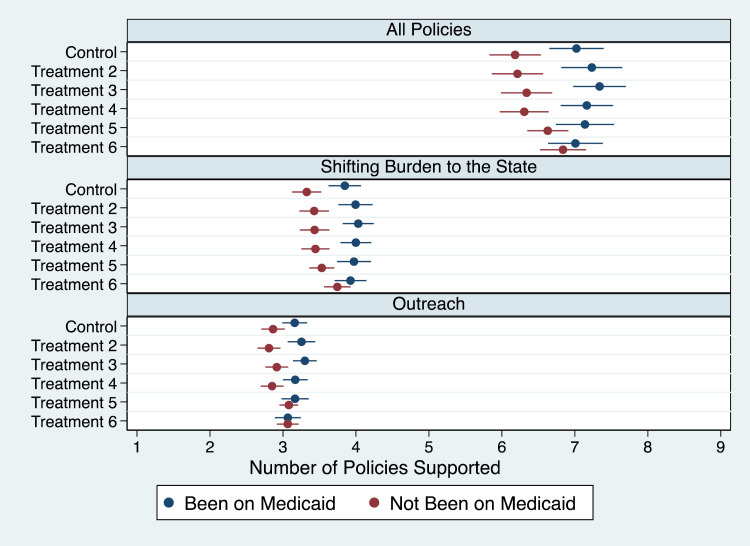
Mean number of policies supported by respondents, by experience with Medicaid program.

Second, we also found our third hypothesis (Figure 3) related to experience with Medicaid generally confirmed. That is, individuals who have personally been enrolled in the Medicaid program were consistently more supportive of reducing administrative burdens than those who have not (*P* < .086). They support between 7.0 and 7.3 policies, while those without any direct exposure only support between 6.2 and 6.8 policies. The only consistent case where we do not find a difference is for the treatment priming respondents for the disproportionate effect of ending the PHE on low-income individuals (*P* = .880). Findings are also in line for the analyses focused on the other two scales (*shifting burdens to the state* and *outreach*) and, again, with minor exceptions, for individual policies.^59^

**Figure 4.**
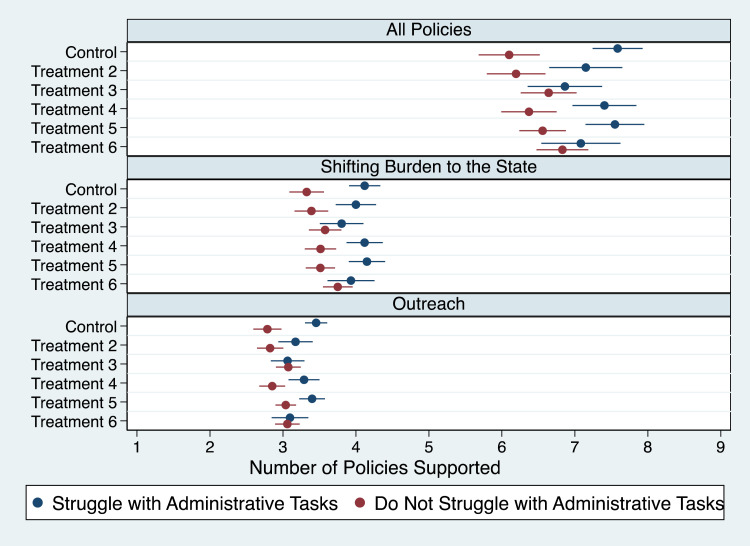
Mean number of policies supported by respondents, by degree of struggle with administrative tasks.

Third, we also found our fourth hypothesis (Figure 4) related to the challenges respondents face in completing administrative tasks generally confirmed. Those who struggle with these types of tasks are generally more supportive of easing the burdens for those affected by the unwinding of the PHE. We found two consistent exceptions for the treatments highlighting the disproportionate effects on racial and ethnic minorities, as well as the disproportionate effect on low-income individuals. Besides these two exceptions, respondents who struggle with administrative tasks supported between 7.2 and 7.6 policies while those who do not supported between 6.1 to 6.6 policies. Our analyses of the two other scales provided analogous results and, again, with minor exceptions, so did our analyses for individual policies.^60^

**Figure 5.**
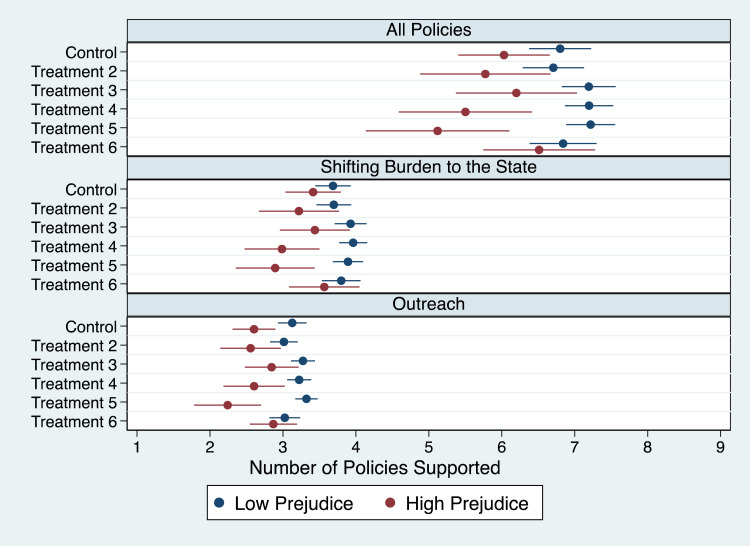
Mean number of policies supported by respondents, by degree of racial prejudice.

Fourth, we found that lower prejudice against minorities was a consistent predictor of support for the reduction in administrative burdens in four of the six treatments (Figure 5). Specifically, those respondents with the lowest levels of prejudice were consistently more supportive of administrative easings by supporting between 6.8 and 7.2 policies as compared with those with the highest levels who supported between 5.1 and 6.2 policies. We did not find statistically significant differences for the treatments highlighting general coverage losses as well as losses focused on low-income individuals. Much of these effects appeared to be the result of differences in policies focused on *outreach,* where we found statistically significant differences for five of the six treatments. Further analysis (see supplementary exhibits A8-10 Appendix) also indicates that these effects are driven by prejudice against Blacks and Hispanics but not Asians. For Blacks, differences are consistently statistically different from each other (*P* < .088), with the exception of the low-income treatment. The same holds for Hispanics (*P* < .098). For Blacks, we also found consistent differences in almost all treatments for both *shifting burdens to the state* and *outreach*, while differences are consistent for Hispanics only for the latter and only present in three of the six cases in the former.

Finally, our analyses generally do not offer consistent evidence that increasing the salience of detrimental effects from unwinding the PHE altered people's views (see supplementary exhibits A11-13 Appendix). A variety of experimental framing treatments that emphasized how burdens might increase coverage losses, impact communities, or affect racial or lower income groups did not have a general effect on the policy preferences of respondents. This may reflect the generally high opposition to burdens we observe among the population, leaving little room for increases when primed for specific detrimental effects.

## Discussion

At a point when state policymakers are considering the degree to which they should make public health insurance more or less accessible as the PHE unwinds, we offer evidence that there is strong support for state actions that ease administrative burdens rather than return to a more onerous status quo ex ante. When given a choice, Americans prefer to see governments take action to make the safety net more accessible, and they do so in an overwhelming fashion. Support for reductions in burden is consistently high and shared among every demographic group (with the exceptions of conservatives for the autorenewal policy option) we considered in our analyses, with three out of four respondents supporting such reductions, on average. Importantly, we provided respondents with policy options that have only limited potential for enrollment fraud. However, such options are real for policymakers, underlining the fact that burden-reduction efforts do not always trigger value tradeoffs.

Nonetheless, support for burden reduction is generally higher for some groups than others, such as liberals and Democrats, those who express less racial prejudice, those who struggle with administrative tasks, or those who have direct personal or family experience with Medicaid. To a degree, such sources of variation help to explain, for example, red state and blue state differences in public health administration. But it would be misleading to overstate the differences, as the results still show that conservatives, those who express racial prejudice, and those without direct experience are generally supportive of burden-reduction efforts.

Our study is not without limitations. First, all standard limitations related to survey research apply. Second, we rely on a one-time sample of respondents queried in late 2022 and early 2023. As far as perceptions are subject to change over time, we are not able to capture those changes. We also relied on an Internet-based survey platform, a common approach in survey research today. The provider of our respondents has been verified as being of high quality and has been used extensively for this type of work. We also employed two attention checks to further improve response quality. Third, we presented respondents with the question of whether they would like to see a policy continued or whether they would like to return to the policy status prior to the pandemic. While this accurately reflects policy reality, alternative presentations could alter responses. Fourth, we deliberately limited our policy choices offered to respondents to policies that carry very limited potential for fraud because they are focused on reducing administrative requirements for beneficiaries or are intended to reduce learning costs. Burden-reduction techniques with more obvious tradeoffs, such as the risk of greater fraud, may not garner as much public support. Last, the survey reflects a normative assumption that those who are legally entitled to health coverage should not lose it for administrative reasons.

As such, the findings suggest a deep well of support for burden-reduction initiatives such as using administrative data to shift burdens away from citizens and onto the state, and better communication and outreach, at least for policies with little potential for fraud. It is worth reiterating that the policies in question do not extend public benefits to additional beneficiaries. They are instead merely efforts to ensure that individuals legally entitled to certain benefits, an estimated 7 million in this case, are able to obtain them. Importantly, these measures also tend to entail large societal benefits. Measures taken during the PHE to facilitate enrollment and maintenance may have contributed to improved program experiences for many beneficiaries, indicating that the reduction in administrative burdens may be an effective tool to support beneficiaries and their families.^61^ Given the general lack of knowledge about the renewal of Medicaid determinations,^20^ the broad support of Americans offers an effective pathway for policymakers to mitigate coverage losses in the wake of the unwinding from the PHE.

While our analyses focused on easing burdens specifically related to the unwinding of the PHE, the results also may indicate a broader opposition to the imposition of burdens generally in health policy and beyond for the Medicaid program as well as other public-assistance programs like the Temporary Assistance for Needy Families (TANF) or SNAP. Future research should seek to establish whether our findings carry over to other programs and outside of a pandemic as well as whether they apply to policies that may hold greater potential for fraudulent or inaccurate enrollment.

## Supplementary Material

qxad001_Supplementary_Data

## References

[1] Halling A, Bækgaard M. Administrative burden in citizen-state interactions: a systematic literature review. OSF Preprints; 2022. https://osf.io/26xdj

[2] Doughty M, Baehler KJ. “Hostages to compliance”: towards a reasonableness test for administrative burdens. Perspecti Public Manage Govern. 2020;3(4):273–287.

[3] Herd P, Moynihan DP. Administrative Burden: Policymaking by Other Means. Russell Sage Foundation; 2018.

[4] Brown JT, Carey G, Malbon E. What is in a form? Examining the complexity of application forms and administrative burden. Aust J Public Adm. 2021;80(4):933–964.

[5] Chudnovsky M, Peeters R. A cascade of exclusion: administrative burdens and access to citizenship in the case of Argentina's national identity document. Int Rev Adm Sci. 2022;88(4):1068–1085.

[6] Nisar MA. Children of a lesser god: administrative burden and social equity in citizen–state interactions. J Public Adm Res Theory. 2018;28(1):104–119.

[7] Peeters R, Trujillo Jimenez H, O’Connor E, Ogarrio Rojas P, Gonzalez Galindo M, Morales Tenorio D. Low-trust bureaucracy: understanding the Mexican bureaucratic experience. Public Adm Dev. 2018;38(2):65–74.

[8] Haeder SF, Gollust SE. From poor to worse: health policy and politics scholars’ assessment of the U.S. COVID-19 response and its implications. World Med Health Policy. 2020;12(4):454–481.

[9] Singer PM, Willison CE, Moore-Petina N, Greer SL. Anatomy of a Failure: COVID-19 in the United States. Coronavirus Politics: The Comparative Politics and Policy of COVID-19. University of Michigan Press; 2021. p. 478–4493.

[10] Public Law No: 116–127. https://www.congress.gov/116/plaws/publ127/PLAW-116publ127.pdf

[11] Tolbert J, Ammula M. 10 Things to Know About the Unwinding of the Medicaid Continuous Enrollment Requirement. Kaiser Family Foundation; 2022.

[12] PHE.gov. Public Health Emergency Declaration Q&As. U.S. Department of Health and Human Services; 2023.

[13] Haeder SF, Sylvester S, Callaghan TH. More than words? How highlighting target populations affects public opinion about the medicaid program. J Health Polit Policy Law. 2023;48(5). 10.1215/03616878-10637708 36995367

[14] Brooks T, Gardner A, Osorio A, et al Medicaid and CHIP Eligibility and Enrollment Policies as of January 2022: Findings from a 50-State Survey. Kaiser Family Foundation; 2022.

[15] McIntyre A, ed. Evidence-based outreach strategies for minimizing coverage loss during unwinding. In: JAMA Health Forum. American Medical Association; 2022. 10.1001/jamahealthforum.2022.3581 36239957

[16] Buettgens M, Green A. The Impact of the COVID-19 Public Health Emergency Expiration on All Types of Health Coverage; 2022. Urban Institute, Washington, DC, 2023. https://www.urban.org/sites/default/files/2022-12/The%20Impact%20of%20the%20COVID-19%20Public%20Health%20Emergency%20Expiration%20on%20All%20Types%20of%20Health%20Coverage_0.pdf

[17] Assistant Secretary for Planning and Evaluation (ASPE). Unwinding the Medicaid Continuous Enrollment Provision: Projected Enrollment Effects and Policy Approaches. U.S. Department of Health and Human Services; 2022.

[18] Arbogast I, Chorniy A, Currie J. Administrative Burdens and Child Medicaid Enrollments. National Bureau of Economic Research; 2022. 10.1086/728170 PMC12129408 40458769

[19] Blum J. Creating a Roadmap for the End of the COVID-19 Public Health Emergency. Centers for Medicare & Medicaid Services; 2022.

[20] Haley JM, Karpman M, Kenney GM, Zuckerman S. Most Adults in Medicaid-Enrolled Families Are Unaware of Medicaid Renewals Resuming in the Future. Urban Institute; 2023.

[21] Fox AM, Stazyk EC, Feng W. Administrative easing: rule reduction and medicaid enrollment. Public Adm Rev. 2020;80(1):104–117.

[22] Kronebusch K, Elbel B. Simplifying children's medicaid and SCHIP. Health Aff (Milwood). 2004;23(3):233–246. 10.1377/hlthaff.23.3.233 15160822

[23] Haeder SF, Sylvester S, Callaghan TH. Lingering legacies: public attitudes about medicaid beneficiaries and work requirements. J Health Polit Policy Law. 2021;46(2):305–355. 32955553 10.1215/03616878-8802198

[24] Herd P, Moynihan D. Administrative burdens in health policy. J Health Hum Services Adm. 2020;43(1).

[25] Heinrich CJ, Camacho S, Henderson SC, Hernández M, Joshi E. Consequences of administrative burden for social safety nets that support the healthy development of children. Jo Policy Anal Manage. 2022;41(1):11–44.

[26] Christensen J, Aarøe L, Baekgaard M, Herd P, Moynihan DP. Human capital and administrative burden: the role of cognitive resources in citizen-state interactions. Public Adm Rev. 2020;80(1):127–136. 32025058 10.1111/puar.13134 PMC6988471

[27] Chudnovsky M, Peeters R. The unequal distribution of administrative burden: a framework and an illustrative case study for understanding variation in people's experience of burdens. Social Policy Adm. 2021;55(4):527–542.

[28] Askelson NM, Brady P, Wright B, Bentler S, Momany ET, Damiano P. Purged from the rolls: a study of medicaid disenrollment in Iowa. Health Equity. 2019;3(1):637–643. 31872169 10.1089/heq.2019.0093 PMC6922057

[29] Haeder SF. Tangled up in side effects. Saving medicaid from work requirements. Milken Inst Rev. 2019;21(2):52–61.

[30] Stuber J, Kronebusch K. Stigma and other determinants of participation in TANF and medicaid. J Policy Anal Manage. 2004;23(3):509–530. 15218879 10.1002/pam.20024

[31] Finkelstein DM, Harding JF, Paulsell D, English B, Hijjawi GR, Ng’andu J. Economic well-being and health: the role of income support programs in promoting health and advancing health equity. Health Aff (Milwood). 2022;41(12):1700–1706. 10.1377/hlthaff.2022.00846 36469819

[32] Hamad R, Gosliner W, Brown EM, et al Understanding take-up of the earned income tax credit among Californians with low income. Health Aff (Milwood). 2022;41(12):1715–1724. 10.1377/hlthaff.2022.00713 36469822

[33] Batra A, Jackson K, Hamad R. Effects of the 2021 expanded child tax credit on adults’ mental health: a quasi-experimental study. Health Aff (Milwood). 2023;42(1):74–82. 10.1377/hlthaff.2022.00733 PMC10089297 36623218

[34] Bækgaard M, Moynihan DP, Thomsen MK. Why do policymakers support administrative burdens? The roles of deservingness, political ideology, and personal experience. J Public Adm Res Theory. 2021;31(1):184–200.

[35] Lowrey A. The Time Tax. The Atlantic. 2021 July 27.

[36] Biden JRJ. Executive Order on Transforming Federal Customer Experience and Service Delivery to Rebuild Trust in Government. The White House; 2021.

[37] Biden JRJ. Continuing to Strengthen Americans’ Access to Affordable, Quality Health Coverage. The White House; 2021.

[38] Office of Management and Budget. Improving Access to Public Benefits Programs Through the Paperwork Reduction Act. Executive Office of the President; 2022.

[39] Coppock A, McClellan OA. Validating the demographic, political, psychological, and experimental results obtained from a new source of online survey respondents. Res Polit. 2019;6(1):2053168018822174.

[40] Herd P, DeLeire T, Harvey H, Moynihan DP. Shifting administrative burden to the state: the case of medicaid take-up. Public Adm Rev. 2013;73(s1):S69–S81.

[41] Halling A, Herd P, Moynihan DP. How difficult should it be? Evidence of burden tolerance from a nationally representative sample. Public Manage Rev. 2022:1–20. 10.1080/14719037.2022.2056910 PMC10805024 38268537

[42] Döring M, Madsen JK. Mitigating psychological costs—the role of citizens’ administrative literacy and social capital. Public Adm Rev. 2022;82(4):671–681.

[43] Kousser T. The politics of discretionary medicaid spending, 1980–1993. J Health Polit Policy Law. 2002;27(4):639–672. 12374292 10.1215/03616878-27-4-639

[44] Olson LK. The Politics of Medicaid. Columbia University Press; 2010.

[45] Leitner JB, Hehman E, Snowden LR. States higher in racial bias spend less on disabled medicaid enrollees. Social Sci Med. 2018;208:150–157. 10.1016/j.socscimed.2018.01.013 29728309

[46] Barrilleaux C, Bernick E. Deservingness, discretion, and the state politics of welfare spending, 1990–96. State Polit Policy Quarterly. 2003;3(1):1–22.

[47] Gilens M. Race and poverty in America: public misperceptions and the American news media. Public Opin Q. 1996;60(4):515–541.

[48] Gilens M. Why Americans Hate Welfare: Race, Media, and the Politics of Antipoverty Policy. University of Chicago Press; 2009.

[49] Snowden L, Graaf G. The “undeserving poor,” racial bias, and medicaid coverage of African Americans. J Black Psychol. 2019;45(3):130–142.

[50] Knowles ED, Lowery BS, Schaumberg RL. Racial prejudice predicts opposition to Obama and his health care reform plan. J Exp Social Psychol. 2010;46(2):420–423.

[51] Tesler M. The spillover of racialization into health care: how President Obama polarized public opinion by racial attitudes and race. Am J Polit Sci. 2012;56(3):690–704.

[52] Haeder SF, Chattopadhyay J. The power of a tweet? Social media, presidential communication, and the politics of health. Pres Stud Q. 2022;52(2):436–473.

[53] Thaler R. Toward a positive theory of consumer choice. J Econ Behav Org. 1980;1(1):39–60.

[54] Kahneman D, Knetsch JL, Thaler RH. Anomalies: the endowment effect, loss aversion, and status quo bias. J Econ Perspect. 1991;5(1):193–206.

[55] Krupnikov Y, Piston S. The political consequences of latino prejudice against blacks. Public Opin Q. 2016;80(2):480–509. 27274574 10.1093/poq/nfw013 PMC4888580

[56] Long S, Freese J. Regression Models for Categorical Dependent Variables Using Stata. 3^rd^ ed. Stata Press; 2014.

[57] Because responses were very similar across different treatments, and in order to facilitate presentation, we pooled the responses across treatments here (*P* < .000). We note in regard of individual policies, we found strong support for the hypothesis across all demographic groups. Indeed, we only identified 2 point estimates (out of 162) that fell below the 50% mark (conservatives for automatic (ex-parte) renewals and those with high levels of racial prejudice for increased outreach efforts). Point estimates are also statistically significant at conventional levels for the vast majority of policies and demographics. Again, conservatives and those with high levels of racial prejudice serve as the exception here.

[58] We also estimated models using respondent partisanship and found analogous results.

[59] We also estimated models using an indicator for whether the respondent indicated whether they ever had a family member enrolled in Medicaid with analogous results.

[60] Davis MH. Measuring individual differences in empathy: evidence for a multidimensional approach. J Personality Social Psychol. 1983; 44(1):113–126. We also estimated models that utilized a previously developed assessment of empathy that we adjusted to assess empathy for individuals struggling with administrative burdens. Our results were analogous. Our findings using the empathy measures is consistently significant across all treatments.

[61] McDaniel M, Karpman M, Kenney GM, Hahn H, Pratt E. Customer Service Experiences and Enrollment Difficulties Vary Widely Across Safety Net Programs. Urban Institute; 2023.

