# Supplementary material for: Most Americans support minimizing administrative burdens for Medicaid recipients as the public health emergency ends
Source: Health Aff Sch. 2023 Jun 20;1(1):qxad001. doi: 10.1093/haschl/qxad001 (PMC10985919; doi:10.1093/haschl/qxad001)
Supplement: qxad001_Supplementary_Data [file qxad001_Supplementary_Data.zip › Appendix R1.docx]

**Appendix**

**Appendix: Exhibit 1:** Distribution of Weights

**
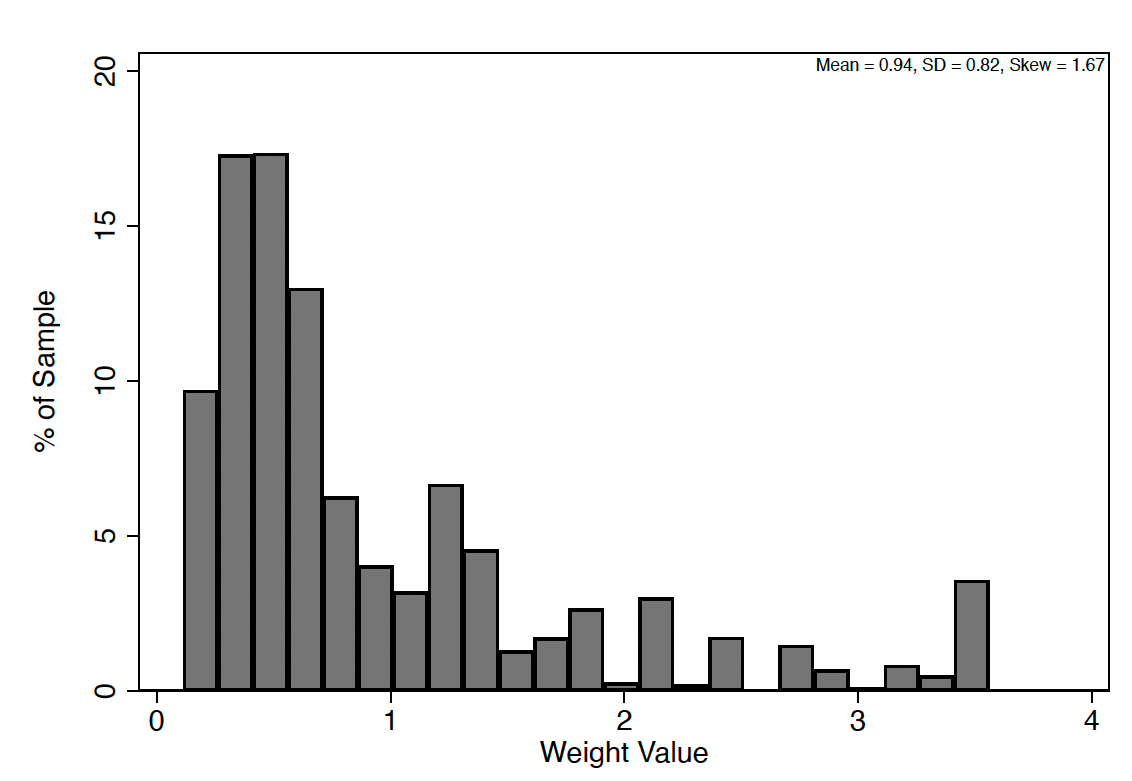
**

**Appendix: Exhibit 2:** Comparison of Raw and Weighted Qualtrics Data to National Benchmarks

| Variable | Survey Data  (Raw) | Survey Data  (Weighted) | Benchmark | Benchmark Source |
| --- | --- | --- | --- | --- |
|  |  |  |  |  |
| Female | 52% | 51% | 51% | CPS |
| College Degree | 39% | 34% | 31% | CPS |
| Black | 11% | 13% | 13% | CPS |
| White | 69% | 64% | 62% | CPS |
| Hispanic | 11% | 15% | 18% | CPS |
| Democrat | 36% | 37% | 34% | ANES (Wgt.) |
| Republican | 29% | 29% | 28% | ANES (Wgt.) |
| Mean Age | 47 | 48 | 47 | ANES (Wgt.) |
| Median Income | $35 – 49,999 | $50 – 74,999 | $55 – 59,999 | ANES (Wgt.) |

Note: Comparison of the data to known population benchmarks. CPS = Current Population Survey. ANES = American National Election Study. Preference is given to CPS considering its sample size and representativeness, but make use of weighted ANES data whenever it was not possible to use CPS (i.e. CPS does not ask questions about Party ID). Weights in column two adjust for gender, education, race, age, and income. Party ID is **not included** in the weighting formula and is shown only due to the potential interests of those who might use or otherwise consume this data. N (Survey Data) = 4,157

**Appendix: Exhibit 3: Survey Questions**

**Questions: Shift Burdens to the State**

1. As the PHE comes to an end, states should use information from other government programs and agencies to automatically renew coverage for eligible beneficiaries when possible
2. As the PHE comes to an end, states should make the renewal process as easy as possible by, for example, sending forms already pre-filled with basic information (like name, address, and family members) with clear instructions, allowing enrollees to renew online or by phone, and minimizing requests to enrollees for additional documents
3. As the PHE comes to an end, states should update beneficiary contact information from other programs that have had more recent contact with enrollees (like food stamps) and from the Post Office address data.
4. As the PHE comes to an end, states should improve account transfer processes with ACA marketplaces (Obamacare) to give them as much relevant information as possible about the enrollee, including updated contact information
5. As the PHE comes to an end, states should make sure that they are adequately staffed to handle the increased workload

**Questions: Outreach and Communication**

1. As the PHE comes to an end, states should use other methods of communication besides mail, such as text and email, to reach people and explain how they can maintain coverage
2. As the PHE comes to an end, states should send current beneficiaries materials and messages that are in plain language, easy to understand, and available in multiple languages
3. As the PHE comes to an end, states should increase outreach efforts such as hiring ACA marketplace (Obamacare) navigators (trained individuals helping people enroll into health insurance) and investing in advertising campaigns to ensure that disenrolled beneficiaries can enroll in alternative coverage without delay
4. As the PHE comes to an end, states should provide clear information on Medicaid termination notices about how to enroll in marketplace coverage and how to get help from an enrollment assister

**Other Survey Questions**

- We hear a lot of talk these days about liberals and conservatives. Here is a five-point scale on which the political views that people might hold are arranged from extremely liberal to extremely conservative. Where would you place yourself on this scale?
  - Extremely Liberal
  - Liberal
  - Moderate; Middle of the road
  - Conservative
  - Extremely conservative
- Generally speaking, do you usually think of yourself as a Republican, a Democrat, and Independent, or what?
  - Republican
  - Democrat
  - Independent
  - Other
- Do you know anyone who has ever been on Medicaid? Mark all that apply.
- In general, how difficult is it for you to complete such administrative tasks as renewing your driver's license, registering your car, or signing up for insurance?
  - Extremely difficult
  - Somewhat difficult
  - Neither easy nor difficult
  - Somewhat easy
  - Extremely easy
- On a scale from 1 to 7, where 1 means HARD-WORKING and 7 means LAZY, where would you rate [racial/ethnic group] in general on this scale?
- On a scale from 1 to 7, where 1 means INTELLIGENT and 7 means UNINTELLIGENT, where would you rate [racial/ethnic group] in general on this scale?
- On a scale from 1 to 7, where 1 means TRUSTWORTHY and 7 means UNTRUSTWORTHY, where would you rate [racial/ethnic group] on this scale?
- Empathy Battery
  - I am very concerned about those most vulnerable to administrative burdens in public assistance programs like Medicaid or food stamps.
    - Strongly disagree
    - Somewhat disagree
    - Neither agree nor disagree
    - Somewhat agree
    - Strongly agree
  - I feel compassion for those most vulnerable to administrative burdens in public assistance programs like Medicaid or food stamps.
    - Strongly disagree
    - Somewhat disagree
    - Neither agree nor disagree
    - Somewhat agree
    - Strongly agree
  - I am quite moved by what can happen to those most vulnerable to administrative burdens in public assistance programs like Medicaid or food stamps.
    - Strongly disagree
    - Somewhat disagree
    - Neither agree nor disagree
    - Somewhat agree
    - Strongly agree

**Appendix: Exhibit 4: Survey Introduction**

The COVID-19 public health emergency (PHE) issued under Trump Administration and extended by President Biden will come to end — possibly in early 2023.

During the PHE, Medicaid agencies cannot disenroll anyone from Medicaid unless they ask to be disenrolled, move out of state, or die.

When the PHE ends, states will resume their regular eligibility reviews of all Medicaid enrollees.

**Appendix: Exhibit 5: Treatments**

1. Coverage Losses: More than 7 million of otherwise eligible beneficiaries are predicted to lose access to public assistance due to difficulty navigating the renewal process and other administrative issues.

2. Racial and ethnic minorities: More than 7 million of otherwise eligible beneficiaries are predicted to lose access to public assistance due to difficulty navigating the renewal process and other administrative issues. Racial and ethnic minorities will be disproportionately affected.

3. Racial inequities and systemic racism: More than 7 million of otherwise eligible beneficiaries are predicted to lose access to public assistance due to difficulty navigating the renewal process and other administrative issues. This will exacerbate racial inequities by furthering systemic racism in the broader healthcare system and society.

4. Community effects: More than 7 million of otherwise eligible beneficiaries are predicted to lose access to public assistance due to difficulty navigating the renewal process and other administrative issues. This may have a negative effect many hospitals and other medical providers have to treat more uninsured patients. Some of them even have to close their business, affecting their entire community.

5. Low-income: More than 7 million of otherwise eligible beneficiaries are predicted to lose access to public assistance due to difficulty navigating the renewal process and other administrative issues. The beneficiaries with the lowest incomes will be disproportionately affected.

**Appendix: Exhibit 6:** Proportion of Respondents Supportive of Reducing Administrative Burdens

|  | Automatic Renewals | Facilitate Renewal | Administrative Capacity | Update Information | Improve Account Transfer | Send Appropriate Materials | Non-traditional Communication | Outreach | Clear Information |
| --- | --- | --- | --- | --- | --- | --- | --- | --- | --- |
| Overall | **0.675** | **0.710** | **0.788** | **0.701** | **0.766** | **0.780** | **0.739** | **0.706** | **0.787** |
|  | . | . | . | . | . | . | . | . | . |
| Female | **0.669** | **0.721** | **0.804** | **0.700** | **0.785** | **0.804** | **0.761** | **0.725** | **0.815** |
| Male | **0.680** | **0.698** | **0.771** | **0.701** | **0.747** | **0.755** | **0.716** | **0.687** | **0.759** |
|  |  |  |  |  |  |  |  |  |  |
| Low Education | **0.696** | **0.712** | **0.790** | **0.693** | **0.758** | **0.784** | **0.742** | **0.724** | **0.786** |
| High Education | **0.690** | **0.726** | **0.800** | **0.737** | **0.794** | **0.787** | **0.762** | **0.709** | **0.803** |
|  | . | . | . | . | . | . | . | . | . |
| Low Income | **0.711** | **0.696** | **0.775** | **0.715** | **0.765** | **0.766** | **0.705** | **0.724** | **0.785** |
| High Income | **0.660** | **0.698** | **0.786** | **0.703** | **0.761** | **0.770** | **0.724** | **0.688** | **0.783** |
|  |  |  |  |  |  |  |  |  |  |
| Democrats | **0.786** | **0.809** | **0.866** | **0.779** | **0.848** | **0.840** | **0.810** | **0.824** | **0.847** |
| Republicans | **0.566** | **0.586** | **0.712** | **0.621** | **0.674** | **0.708** | **0.643** | **0.574** | **0.716** |
|  | . | . | . | . | . | . | . | . | . |
| Liberals | **0.831** | **0.823** | **0.878** | **0.817** | **0.870** | **0.870** | **0.839** | **0.846** | **0.893** |
| Conservatives | 0.528 | **0.567** | **0.689** | **0.592** | **0.657** | **0.690** | **0.635** | **0.545** | **0.692** |
|  | . | . | . | . | . | . | . | . | . |
| Been on Medicaid | **0.778** | **0.782** | **0.843** | **0.755** | **0.800** | **0.814** | **0.788** | **0.769** | **0.815** |
| Not been on Medicaid | **0.623** | **0.674** | **0.760** | **0.673** | **0.749** | **0.763** | **0.715** | **0.675** | **0.773** |
|  | . | . | . | . | . | . | . | . | . |
| Struggle with Tasks | **0.798** | **0.773** | **0.830** | **0.781** | **0.827** | **0.821** | **0.808** | **0.777** | **0.839** |
| Doesn't Struggle with Tasks | **0.633** | **0.682** | **0.777** | **0.677** | **0.747** | **0.764** | **0.714** | **0.685** | **0.774** |
|  | . | . | . | . | . | . | . | . | . |
| Low Prejudice | **0.726** | **0.753** | **0.802** | **0.742** | **0.808** | **0.801** | **0.779** | **0.767** | **0.821** |
| High Prejudice | **0.640** | **0.629** | **0.711** | **0.614** | **0.683** | **0.721** | **0.615** | **0.609** | **0.706** |

Note: Results statistically larger than 0.500 at p<0.05 highlighted in **bold**

**Appendix: Exhibit 7**: Support for Policies Reducing Administrative Burdens Transitioning out of the Public Health Emergency

|  |
| --- |
|  |
|  |

**Appendix: Exhibit 8**: Comparison of Variables of Interest for Various Treatments, Overall

| Comparison | |  | Mean 1 | Mean 2 | Delta | P-value |
| --- | --- | --- | --- | --- | --- | --- |
| **Democrats v. Republicans** | | Control | 7.204 | 5.431 | -1.773 | 0.000 |
|  |  | Coverage Losses | 7.444 | 5.613 | -1.832 | 0.000 |
|  |  | Racial& Ethnic Minorites | 7.147 | 6.256 | -0.890 | 0.025 |
|  |  | Inequities & Systemic Racism | 7.530 | 5.656 | -1.874 | 0.000 |
|  |  | Community Effects | 7.348 | 5.952 | -1.396 | 0.000 |
|  |  | Low Income | 7.803 | 5.863 | -1.939 | 0.000 |
|  |  |  |  |  |  |  |
| **Liberals v. Conservatives** | | Control | 7.511 | 5.259 | -2.252 | 0.000 |
|  |  | Coverage Losses | 7.909 | 5.297 | -2.612 | 0.000 |
|  |  | Racial& Ethnic Minorites | 7.491 | 6.077 | -1.413 | 0.001 |
|  |  | Inequities & Systemic Racism | 7.897 | 5.442 | -2.456 | 0.000 |
|  |  | Community Effects | 7.432 | 5.815 | -1.617 | 0.000 |
|  |  | Low Income | 7.722 | 5.693 | -2.029 | 0.000 |
|  |  |  |  |  |  |  |
| **Medicaid Connection** | | Control | 7.022 | 6.181 | -0.840 | 0.007 |
| **Self vs. Not** | | Coverage Losses | 7.233 | 6.213 | -1.019 | 0.002 |
|  |  | Racial& Ethnic Minorites | 7.339 | 6.339 | -0.999 | 0.001 |
|  |  | Inequities & Systemic Racism | 7.164 | 6.307 | -0.858 | 0.004 |
|  |  | Community Effects | 7.139 | 6.629 | -0.510 | 0.086 |
|  |  | Low Income | 7.008 | 6.838 | -0.170 | 0.569 |
|  |  |  |  |  |  |  |
| **Medicaid Connection** | | Control | 6.682 | 6.025 | -0.657 | 0.076 |
| **Family vs. Not** | | Coverage Losses | 6.912 | 5.732 | -1.180 | 0.002 |
|  |  | Racial& Ethnic Minorites | 6.953 | 6.054 | -0.900 | 0.018 |
|  |  | Inequities & Systemic Racism | 6.830 | 6.139 | -0.691 | 0.053 |
|  |  | Community Effects | 6.995 | 6.316 | -0.679 | 0.033 |
|  |  | Low Income | 6.910 | 6.862 | -0.048 | 0.880 |
|  |  |  |  |  |  |  |
| **Administrative Tasks** | | Control | 7.586 | 6.100 | -1.486 | 0.000 |
| **Difficult v. Easy** | | Coverage Losses | 7.150 | 6.195 | -0.956 | 0.014 |
|  |  | Racial& Ethnic Minorites | 6.864 | 6.641 | -0.223 | 0.566 |
|  |  | Inequities & Systemic Racism | 7.405 | 6.371 | -1.034 | 0.003 |
|  |  | Community Effects | 7.550 | 6.559 | -0.991 | 0.002 |
|  |  | Low Income | 7.082 | 6.830 | -0.252 | 0.522 |
|  |  |  |  |  |  |  |
| **Low v. High Empathy** | | Control | 5.121 | 7.780 | 2.659 | 0.000 |
|  |  | Coverage Losses | 5.005 | 8.320 | 3.315 | 0.000 |
|  |  | Racial& Ethnic Minorites | 5.452 | 8.084 | 2.633 | 0.000 |
|  |  | Inequities & Systemic Racism | 5.397 | 8.055 | 2.658 | 0.000 |
|  |  | Community Effects | 5.399 | 7.895 | 2.496 | 0.000 |
|  |  | Low Income | 5.305 | 8.027 | 2.722 | 0.000 |
|  |  |  |  |  |  |  |
| **Prejudice** | | Control | 6.800 | 6.030 | -0.770 | 0.095 |
| **Low v. High Minority** | | Coverage Losses | 6.707 | 5.775 | -0.933 | 0.120 |
|  |  | Racial& Ethnic Minorites | 7.193 | 6.201 | -0.992 | 0.073 |
|  |  | Inequities & Systemic Racism | 7.197 | 5.502 | -1.695 | 0.004 |
|  |  | Community Effects | 7.218 | 5.120 | -2.097 | 0.001 |
|  |  | Low Income | 6.841 | 6.512 | -0.330 | 0.544 |
|  |  |  |  |  |  |  |
| **Prejudice** | | Control | 6.832 | 6.058 | -0.774 | 0.088 |
| **Low v. High Black** | | Coverage Losses | 6.829 | 5.972 | -0.857 | 0.078 |
|  |  | Racial& Ethnic Minorites | 7.232 | 6.202 | -1.030 | 0.035 |
|  |  | Inequities & Systemic Racism | 7.099 | 5.799 | -1.300 | 0.003 |
|  |  | Community Effects | 7.347 | 6.075 | -1.272 | 0.005 |
|  |  | Low Income | 7.012 | 6.342 | -0.671 | 0.124 |
|  |  |  |  |  |  |  |
| **Prejudice** | | Control | 6.702 | 6.285 | -0.417 | 0.315 |
| **Low v. High Asian** | | Coverage Losses | 6.628 | 6.566 | -0.063 | 0.883 |
|  |  | Racial& Ethnic Minorites | 6.832 | 6.439 | -0.393 | 0.341 |
|  |  | Inequities & Systemic Racism | 6.944 | 6.114 | -0.830 | 0.034 |
|  |  | Community Effects | 7.164 | 6.603 | -0.561 | 0.120 |
|  |  | Low Income | 6.881 | 6.971 | 0.090 | 0.807 |
|  |  |  |  |  |  |  |
| **Prejudice** | | Control | 6.667 | 5.916 | -0.751 | 0.098 |
| **Low v. High Hispanic** | | Coverage Losses | 6.724 | 5.087 | -1.637 | 0.012 |
|  |  | Racial& Ethnic Minorites | 7.104 | 6.257 | -0.847 | 0.100 |
|  |  | Inequities & Systemic Racism | 7.169 | 5.684 | -1.485 | 0.013 |
|  |  | Community Effects | 7.057 | 5.680 | -1.376 | 0.004 |
|  |  | Low Income | 6.731 | 7.145 | 0.415 | 0.377 |
|  |  |  |  |  |  |  |
| **Low v. High Knowledge** | | Control | 5.998 | 7.400 | 1.402 | 0.010 |
|  |  | Coverage Losses | 6.640 | 7.528 | 0.888 | 0.045 |
|  |  | Racial& Ethnic Minorites | 4.801 | 7.725 | 2.924 | 0.000 |
|  |  | Inequities & Systemic Racism | 6.544 | 7.294 | 0.750 | 0.085 |
|  |  | Community Effects | 5.017 | 7.381 | 2.364 | 0.000 |
|  |  | Low Income | 5.846 | 7.889 | 2.044 | 0.000 |

**Appendix: Exhibit 9**: Comparison of Variables of Interest for Various Treatments, Shifting Burden to State

| Comparison | |  | Mean 1 | Mean 2 | Delta | P-value |
| --- | --- | --- | --- | --- | --- | --- |
| **Democrats v. Republicans** | | Control | 3.900 | 2.971 | -0.928 | 0.000 |
|  |  | Coverage Losses | 4.104 | 3.092 | -1.012 | 0.000 |
|  |  | Racial& Ethnic Minorites | 3.891 | 3.414 | -0.477 | 0.038 |
|  |  | Inequities & Systemic Racism | 4.225 | 3.042 | -1.183 | 0.000 |
|  |  | Community Effects | 4.070 | 3.221 | -0.849 | 0.000 |
|  |  | Low Income | 4.332 | 3.207 | -1.125 | 0.000 |
|  |  |  |  |  |  |  |
| **Liberals v. Conservatives** | | Control | 4.099 | 2.846 | -1.253 | 0.000 |
|  |  | Coverage Losses | 4.369 | 2.900 | -1.469 | 0.000 |
|  |  | Racial& Ethnic Minorites | 4.086 | 3.337 | -0.749 | 0.003 |
|  |  | Inequities & Systemic Racism | 4.401 | 2.977 | -1.424 | 0.000 |
|  |  | Community Effects | 4.063 | 3.105 | -0.958 | 0.000 |
|  |  | Low Income | 4.308 | 3.047 | -1.261 | 0.000 |
|  |  |  |  |  |  |  |
| **Medicaid Connection** | | Control | 3.847 | 3.325 | -0.522 | 0.004 |
| **Self vs. Not** | | Coverage Losses | 3.995 | 3.428 | -0.567 | 0.003 |
|  |  | Racial& Ethnic Minorites | 4.032 | 3.433 | -0.599 | 0.001 |
|  |  | Inequities & Systemic Racism | 3.999 | 3.445 | -0.554 | 0.001 |
|  |  | Community Effects | 3.973 | 3.532 | -0.441 | 0.012 |
|  |  | Low Income | 3.926 | 3.744 | -0.182 | 0.293 |
|  |  | . | . | . | . | . |
| **Medicaid Connection** | | Control | 3.636 | 3.225 | -0.412 | 0.052 |
| **Family vs. Not** | | Coverage Losses | 3.816 | 3.164 | -0.652 | 0.003 |
|  |  | Racial& Ethnic Minorites | 3.806 | 3.254 | -0.552 | 0.013 |
|  |  | Inequities & Systemic Racism | 3.783 | 3.338 | -0.446 | 0.029 |
|  |  | Community Effects | 3.790 | 3.391 | -0.399 | 0.035 |
|  |  | Low Income | 3.795 | 3.828 | 0.033 | 0.857 |
|  |  |  |  |  |  |  |
| **Administrative Tasks** | | Control | 4.120 | 3.325 | -0.795 | 0.000 |
| **Difficult v. Easy** | | Coverage Losses | 3.999 | 3.390 | -0.610 | 0.005 |
|  |  | Racial& Ethnic Minorites | 3.804 | 3.578 | -0.226 | 0.318 |
|  |  | Inequities & Systemic Racism | 4.119 | 3.514 | -0.605 | 0.002 |
|  |  | Community Effects | 4.151 | 3.513 | -0.638 | 0.001 |
|  |  | Low Income | 3.932 | 3.751 | -0.181 | 0.434 |
|  |  |  |  |  |  |  |
| **Low v. High Empathy** | | Control | 2.772 | 4.241 | 1.469 | 0.000 |
|  |  | Coverage Losses | 2.739 | 4.612 | 1.874 | 0.000 |
|  |  | Racial& Ethnic Minorites | 2.944 | 4.483 | 1.539 | 0.000 |
|  |  | Inequities & Systemic Racism | 3.007 | 4.441 | 1.433 | 0.000 |
|  |  | Community Effects | 2.824 | 4.312 | 1.489 | 0.000 |
|  |  | Low Income | 2.934 | 4.470 | 1.535 | 0.000 |
|  |  |  |  |  |  |  |
| **Prejudice** | | Control | 3.686 | 3.414 | -0.273 | 0.320 |
| **Low v. High Minority** | | Coverage Losses | 3.697 | 3.219 | -0.478 | 0.189 |
|  |  | Racial& Ethnic Minorites | 3.927 | 3.438 | -0.489 | 0.127 |
|  |  | Inequities & Systemic Racism | 3.963 | 2.986 | -0.976 | 0.003 |
|  |  | Community Effects | 3.892 | 2.894 | -0.998 | 0.005 |
|  |  | Low Income | 3.799 | 3.566 | -0.233 | 0.488 |
|  |  |  |  |  |  |  |
| **Prejudice** | | Control | 3.743 | 3.308 | -0.436 | 0.096 |
| **Low v. High Black** | | Coverage Losses | 3.768 | 3.327 | -0.441 | 0.128 |
|  |  | Racial& Ethnic Minorites | 3.981 | 3.400 | -0.581 | 0.036 |
|  |  | Inequities & Systemic Racism | 3.898 | 3.187 | -0.711 | 0.006 |
|  |  | Community Effects | 4.000 | 3.285 | -0.715 | 0.007 |
|  |  | Low Income | 3.885 | 3.449 | -0.436 | 0.091 |
|  |  |  |  |  |  |  |
| **Prejudice** | | Control | 3.644 | 3.448 | -0.196 | 0.404 |
| **Low v. High Asian** | | Coverage Losses | 3.643 | 3.654 | 0.011 | 0.965 |
|  |  | Racial& Ethnic Minorites | 3.712 | 3.483 | -0.229 | 0.341 |
|  |  | Inequities & Systemic Racism | 3.875 | 3.381 | -0.494 | 0.029 |
|  |  | Community Effects | 3.862 | 3.651 | -0.211 | 0.325 |
|  |  | Low Income | 3.760 | 3.847 | 0.087 | 0.680 |
|  |  |  |  |  |  |  |
| **Prejudice** | | Control | 3.635 | 3.281 | -0.353 | 0.181 |
| **Low v. High Hispanic** | | Coverage Losses | 3.696 | 2.970 | -0.726 | 0.060 |
|  |  | Racial& Ethnic Minorites | 3.890 | 3.495 | -0.396 | 0.186 |
|  |  | Inequities & Systemic Racism | 3.954 | 3.024 | -0.930 | 0.005 |
|  |  | Community Effects | 3.817 | 3.147 | -0.671 | 0.017 |
|  |  | Low Income | 3.751 | 3.951 | 0.201 | 0.489 |
|  |  |  |  |  |  |  |
| **Low v. High Knowledge** | | Control | 3.333 | 3.990 | 0.657 | 0.035 |
|  |  | Coverage Losses | 3.618 | 4.156 | 0.538 | 0.048 |
|  |  | Racial& Ethnic Minorites | 2.718 | 4.238 | 1.520 | 0.000 |
|  |  | Inequities & Systemic Racism | 3.552 | 4.048 | 0.496 | 0.053 |
|  |  | Community Effects | 2.714 | 3.997 | 1.283 | 0.000 |
|  |  | Low Income | 3.193 | 4.342 | 1.149 | 0.000 |

**Appendix: Exhibit 10**: Comparison of Variables of Interest for Various Treatments, Outreach

| Comparison | |  | Mean 1 | Mean 2 | Delta | P-value |
| --- | --- | --- | --- | --- | --- | --- |
| **Democrats v. Republicans** | | Control | 3.296 | 2.476 | -0.820 | 0.000 |
|  |  | Coverage Losses | 3.343 | 2.550 | -0.793 | 0.000 |
|  |  | Racial& Ethnic Minorites | 3.266 | 2.857 | -0.409 | 0.021 |
|  |  | Inequities & Systemic Racism | 3.306 | 2.612 | -0.694 | 0.000 |
|  |  | Community Effects | 3.278 | 2.711 | -0.567 | 0.001 |
|  |  | Low Income | 3.434 | 2.657 | -0.778 | 0.000 |
|  |  | . | . | . | . | . |
| **Liberals v. Conservatives** | | Control | 3.412 | 2.435 | -0.976 | 0.000 |
|  |  | Coverage Losses | 3.550 | 2.431 | -1.118 | 0.000 |
|  |  | Racial& Ethnic Minorites | 3.411 | 2.757 | -0.654 | 0.000 |
|  |  | Inequities & Systemic Racism | 3.498 | 2.469 | -1.029 | 0.000 |
|  |  | Community Effects | 3.374 | 2.694 | -0.680 | 0.000 |
|  |  | Low Income | 3.429 | 2.645 | -0.784 | 0.000 |
|  |  |  |  |  |  |  |
| **Medicaid Connection** | | Control | 3.161 | 2.864 | -0.297 | 0.037 |
| **Self vs. Not** | | Coverage Losses | 3.254 | 2.808 | -0.446 | 0.003 |
|  |  | Racial& Ethnic Minorites | 3.300 | 2.915 | -0.385 | 0.005 |
|  |  | Inequities & Systemic Racism | 3.168 | 2.850 | -0.318 | 0.024 |
|  |  | Community Effects | 3.165 | 3.080 | -0.085 | 0.538 |
|  |  | Low Income | 3.066 | 3.065 | -0.001 | 0.995 |
|  |  |  |  |  |  |  |
| **Medicaid Connection** | | Control | 3.041 | 2.803 | -0.239 | 0.158 |
| **Family vs. Not** | | Coverage Losses | 3.114 | 2.602 | -0.512 | 0.003 |
|  |  | Racial& Ethnic Minorites | 3.146 | 2.822 | -0.324 | 0.055 |
|  |  | Inequities & Systemic Racism | 3.050 | 2.776 | -0.274 | 0.100 |
|  |  | Community Effects | 3.193 | 2.910 | -0.283 | 0.055 |
|  |  | Low Income | 3.079 | 3.037 | -0.042 | 0.789 |
|  |  |  |  |  |  |  |
| **Administrative Tasks** | | Control | 3.454 | 2.786 | -0.668 | 0.000 |
| **Difficult v. Easy** | | Coverage Losses | 3.172 | 2.824 | -0.348 | 0.053 |
|  |  | Racial& Ethnic Minorites | 3.064 | 3.073 | 0.009 | 0.957 |
|  |  | Inequities & Systemic Racism | 3.288 | 2.853 | -0.436 | 0.010 |
|  |  | Community Effects | 3.399 | 3.038 | -0.361 | 0.009 |
|  |  | Low Income | 3.096 | 3.060 | -0.036 | 0.846 |
|  |  |  |  |  |  |  |
| **Low v. High Empathy** | | Control | 2.337 | 3.535 | 1.198 | 0.000 |
|  |  | Coverage Losses | 2.272 | 3.717 | 1.445 | 0.000 |
|  |  | Racial& Ethnic Minorites | 2.509 | 3.616 | 1.106 | 0.000 |
|  |  | Inequities & Systemic Racism | 2.373 | 3.616 | 1.243 | 0.000 |
|  |  | Community Effects | 2.551 | 3.588 | 1.037 | 0.000 |
|  |  | Low Income | 2.332 | 3.549 | 1.217 | 0.000 |
|  |  |  |  |  |  |  |
| **Prejudice** | | Control | 3.128 | 2.604 | -0.524 | 0.015 |
| **Low v. High Minority** | | Coverage Losses | 3.013 | 2.556 | -0.457 | 0.099 |
|  |  | Racial& Ethnic Minorites | 3.274 | 2.846 | -0.429 | 0.080 |
|  |  | Inequities & Systemic Racism | 3.223 | 2.605 | -0.618 | 0.024 |
|  |  | Community Effects | 3.322 | 2.242 | -1.080 | 0.000 |
|  |  | Low Income | 3.025 | 2.868 | -0.157 | 0.504 |
|  |  | . | . | . | . | . |
| **Prejudice** | | Control | 3.104 | 2.741 | -0.363 | 0.082 |
| **Low v. High Black** | | Coverage Losses | 3.079 | 2.643 | -0.436 | 0.050 |
|  |  | Racial& Ethnic Minorites | 3.256 | 2.848 | -0.408 | 0.064 |
|  |  | Inequities & Systemic Racism | 3.188 | 2.653 | -0.535 | 0.009 |
|  |  | Community Effects | 3.343 | 2.789 | -0.554 | 0.008 |
|  |  | Low Income | 3.104 | 2.854 | -0.250 | 0.215 |
|  |  | . | . | . | . | . |
| **Prejudice** | | Control | 3.069 | 2.826 | -0.244 | 0.196 |
| **Low v. High Asian** | | Coverage Losses | 2.989 | 2.955 | -0.034 | 0.858 |
|  |  | Racial& Ethnic Minorites | 3.128 | 2.960 | -0.168 | 0.360 |
|  |  | Inequities & Systemic Racism | 3.069 | 2.755 | -0.314 | 0.085 |
|  |  | Community Effects | 3.283 | 2.951 | -0.333 | 0.043 |
|  |  | Low Income | 3.087 | 3.086 | -0.001 | 0.997 |
|  |  | . | . | . | . | . |
| **Prejudice** | | Control | 3.039 | 2.620 | -0.419 | 0.059 |
| **Low v. High Hispanic** | | Coverage Losses | 3.031 | 2.118 | -0.913 | 0.002 |
|  |  | Racial& Ethnic Minorites | 3.219 | 2.831 | -0.388 | 0.089 |
|  |  | Inequities & Systemic Racism | 3.205 | 2.724 | -0.482 | 0.080 |
|  |  | Community Effects | 3.235 | 2.539 | -0.696 | 0.003 |
|  |  | Low Income | 2.944 | 3.194 | 0.251 | 0.202 |
|  |  |  |  |  |  |  |
| **Low v. High Knowledge** | | Control | 2.651 | 3.389 | 0.738 | 0.003 |
|  |  | Coverage Losses | 3.126 | 3.376 | 0.250 | 0.206 |
|  |  | Racial& Ethnic Minorites | 2.083 | 3.499 | 1.417 | 0.000 |
|  |  | Inequities & Systemic Racism | 2.888 | 3.257 | 0.369 | 0.079 |
|  |  | Community Effects | 2.315 | 3.385 | 1.070 | 0.000 |
|  |  | Low Income | 2.617 | 3.510 | 0.893 | 0.000 |

**Appendix: Exhibit 11**: Comparison of Treatments, Overall

|  |  |  | Mean 1 | Mean 2 | Delta | P-value |
| --- | --- | --- | --- | --- | --- | --- |
|  |  | Control | 6.468 | . | . | . |
| Control | vs | Coverage Losses | 6.468 | 6.527 | 0.058 | 0.803 |
| Control | vs | Racial& Ethnic Minorites | 6.468 | 6.708 | 0.240 | 0.295 |
| Control | vs | Inequities & Systemic Racism | 6.468 | 6.593 | 0.125 | 0.579 |
| Control | vs | Community Effects | 6.468 | 6.787 | 0.319 | 0.137 |
| Control | vs | Low Income | 6.468 | 6.895 | 0.427 | 0.053 |

**Appendix: Exhibit 12**: Comparison of Treatments, Shifting Burden to State

|  |  |  | Mean 1 | Mean 2 | Delta | P-value |
| --- | --- | --- | --- | --- | --- | --- |
|  |  | Control | 3.503 | . | . | . |
| Control | vs | Coverage Losses | 3.503 | 3.603 | 0.100 | 0.456 |
| Control | vs | Racial& Ethnic Minorites | 3.503 | 3.657 | 0.153 | 0.246 |
| Control | vs | Inequities & Systemic Racism | 3.503 | 3.630 | 0.127 | 0.330 |
| Control | vs | Community Effects | 3.503 | 3.669 | 0.165 | 0.190 |
| Control | vs | Low Income | 3.503 | 3.805 | 0.302 | 0.018 |

**Appendix: Exhibit 13**: Comparison of Treatments, Outreach

|  |  |  | Mean 1 | Mean 2 | Delta | P-value |
| --- | --- | --- | --- | --- | --- | --- |
|  |  | Control | 2.964 | . | . | . |
| Control | vs | Coverage Losses | 2.964 | 2.946 | -0.018 | 0.863 |
| Control | vs | Racial& Ethnic Minorites | 2.964 | 3.057 | 0.093 | 0.369 |
| Control | vs | Inequities & Systemic Racism | 2.964 | 2.956 | -0.008 | 0.938 |
| Control | vs | Community Effects | 2.964 | 3.107 | 0.142 | 0.145 |
| Control | vs | Low Income | 2.964 | 3.066 | 0.101 | 0.322 |
